# Increased Risk of COVID-19 in Patients with Diabetes Mellitus—Current Challenges in Pathophysiology, Treatment and Prevention

**DOI:** 10.3390/ijerph19116555

**Published:** 2022-05-27

**Authors:** Tomasz Gęca, Kamila Wojtowicz, Paweł Guzik, Tomasz Góra

**Affiliations:** 1 Chair and Department of Obstetrics and Pathology of Pregnancy, Medical University of Lublin, 20-081 Lublin, Poland; 2 Clinical Department of Gynecology and Obstetrics, John Paul’s 2nd Municipal Hospital, 35-241 Rzeszow, Poland; guccii@poczta.onet.pl (K.W.); pawelguzik@gmail.com (P.G.); 3 Chair and Department of Gynecology and Obstetrics, Jagiellonian University Medical College, 31-501 Krakow, Poland

**Keywords:** COVID-19, diabetes mellitus, SARS-CoV-2, oxidative stress, angiotensin converting enzyme-2

## Abstract

Coronavirus disease—COVID-19 (coronavirus disease 2019) has become the cause of the global pandemic in the last three years. Its etiological factor is SARS-CoV-2 (Severe Acute Respiratory Syndrome Coronavirus type 2). Patients with diabetes (DM—diabetes mellitus), in contrast to healthy people not suffering from chronic diseases, are characterised by higher morbidity and mortality due to COVID-19. Patients who test positive for SARCoV-2 are at higher risk of developing hyperglycaemia. In this paper, we present, analyse and summarize the data on possible mechanisms underlying the increased susceptibility and mortality of patients with diabetes mellitus in the case of SARS-CoV-2 infection. However, further research is required to determine the optimal therapeutic management of patients with diabetes and COVID-19.

## 1. Introduction

**Figure 1:**
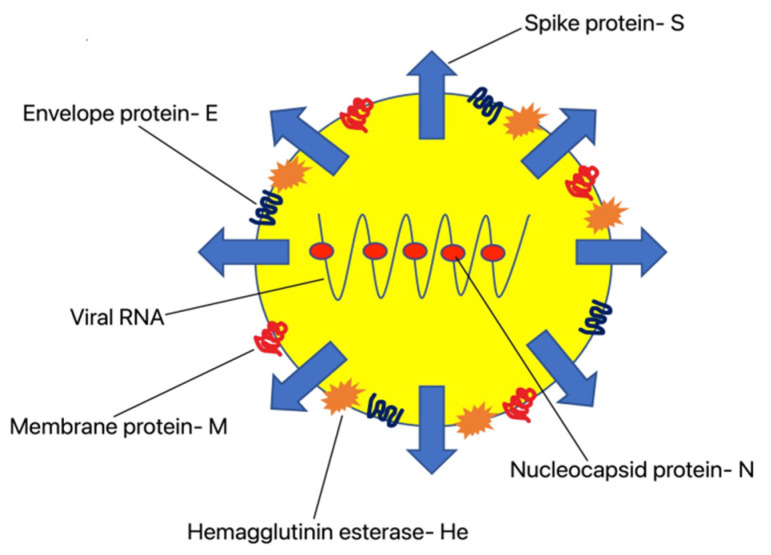
A model of COVID-19 depicting structural elements of the virion.

The World Health Organization (WHO) has classified the 2019 Coronavirus pandemic (COVID-19) as a global health emergency [1]. The virus causes acute respiratory distress syndrome. So far our knowledge of this disease has been limited. The new beta-coronavirus was first introduced in Wuhan, China in 2019 and then spread worldwide. SARS-CoV-2 is an RNA virus with genomes ranging from 26,000 to 32,000 base pairs [2]. The capsid contains four structural proteins: spike (S), nucleocapsid (N), membrane (M) and envelope (E). In their research, Walls et al. showed that the nucleic acid and developmental N acid are under the formed lipid novel solution of the SARS-CoV-2 virion [3]. Under an electron microscope, the S looks like a corona and thus its name: coronavirus. The S protein consists of two domains. The binding of the receptor for angiotenin-converting enzyme-type 2 (ACE-2) takes place in the upper lobular domain, which initiates a viral entry into cells. The downstream domain of the S protein is involved in the virus attachment to the host-cell membrane [4]. The function of the M protein is to bind the viral genome to the inner surface of the host-cell membrane. The C-terminal domain of the transmembrane proteins contacts the viral N protein, playing an important role in its life cycle [5]. The scheme showing the model of COVID-19 virus is presented in Figure 1.

**Figure 2:**
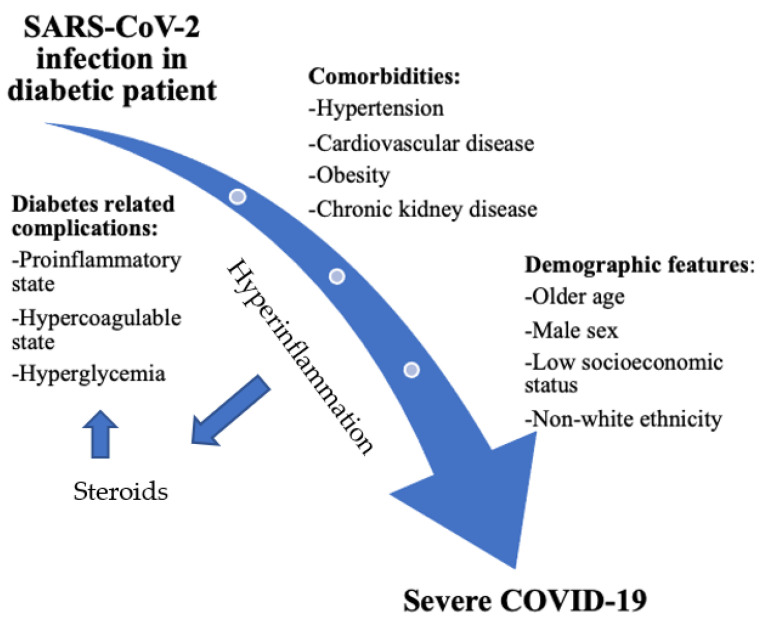
The relationship between SARS-CoV-2 infection, COVID-19 and diabetes. In a diabetic patient, the presence of diabetes-related complications, associated comorbidities and certain demographic features may worsen the prognosis. Hyperglycemia is a strong risk factor for a severe course of COVID-19. Hyperinflammation associated with severe COVID-19 treated with steroids can worsen hyperglycemia.

In the SARS-CoV-2 replication cycle, several key stages can be distinguished: attachment and entry into the cell, transcription of the viral replicase, genome replication, translation of structural proteins and release of new virions [2]. The analysis of over 300,000 SARS-CoV-2 genomes, performed less than a year ago by Rochman et al., showed some kind of an adaptive evolution of this virus which affects not only the nucleocapsid itself, but also many regions in the viral S protein [6]. Therefore, based on mutations in the RBD region of the viral S protein, a total of seven mutant variants of SARS-CoV-2 have been described: alpha (B.1.1.7), beta (B.1.351), gamma (P.1), delta (B.1.617.2), epsilon (B.1.427), kappa (B.1.617.1), and the Omicron variant (B.1.1.529). Based on microscale thermophoresis analyses on the S-protein in SARS-CoV-2, it has been demonstrated that the alpha, beta, gamma and delta variants are characterised by the strongest interaction with the ACE2 receptor [7]. It is also worth emphasizing that the Omicron variant is typical due to its ability to avoid the human immune system [8]. The mortality rate for COVID-19 infection has been estimated at 0.5–1.0% [9,10,11]. The proportion of diabetics among COVID-19-positive patients varies according to the regions in the world. For example, in Italy as many as 36% of those seriously ill, having a positive result from a COVID-19 test, were burdened with diabetes [12], and in the United States, this same phenomenon was noted in as many as 58% of patients [13]. The vast majority of infected patients present a mild form of COVID-19, but some develop a severe form of infection that can be fatal. Factors that influence the development of severe disease are: older age, comorbidities, difficult socio-economic conditions and occupational risk (increased epidemiological treat). The most frequently quoted age limit above which there is an increased risk of serious complications is 60 years and above [14,15]. Regardless of age, patients with comorbid chronic conditions that compromise the immune system are at a higher risk of becoming infected with SARS CoV-2. These diseases mainly include: diabetes [16], renal failure [17], chronic heart and brain diseases [18,19], malignant neoplasms [14], immune disorders [15] and obesity [20]. In recent studies, a significantly higher risk of in-hospital deaths has been noted due to COVID-19 in diabetic patients than in those without glucose tolerance impairment (HR = 2.36) [14]. Interestingly, proper glycaemic control before admission to hospital, confirmed by a glycosylated haemoglobin test (HbA1c), was not associated with improved outcomes in the treatment of infections among these patients [21]. The scheme showing the interrelationship between SARS-CoV-2 infection, diabetes mellitus and risk factors of severe COVID-19 is presented in Figure 2.

## 2. Materials and Methods

### 2.1. Increased Susceptibility to COVID-19 in Diabetics

According to Gregory et al., patients with type 2 (DM2) as well as type 1 diabetes (DM1) are characterised by increased susceptibility to the development of severe disease caused by COVID-19 compared to healthy patients. In this study, patients with DM1 and DM2 had a similar adjusted odds ratio (OR) for hospitalisation rates (3.90 for DM1 vs. 3.36 for DM2) and disease severity (3.35 vs. 3.42) [22]. In Table 1, a detailed analysis is provided on the literature in different populations of patients with type 1 and type 2 diabetes mellitus. In it, an increased risk of death in patients with uncontrolled diabetes and high fasting blood glucose is shown.

An increased risk of SARS-CoV-2 infection in diabetic patients may be associated with drug-induced expression of angiotensin converting enzyme type 2 (ACE-2) receptor. Some hypoglycaemic drugs, such as glucagon-like peptide-1 agonists and thiazolidinediones, overexpress ACE-2 receptor and thus, facilitate the entry of SARS-CoV-2 into cells. Similarly, sodium-glucose co-transporter-2 inhibitors (SGLT2i) also facilitate viral entry into cells, indirectly overexpressing ACE-2, mainly when used together with angiotensin converting enzyme inhibitors (ACE-I). Moreover, treating patients with new hypoglycaemic drugs (such as glucagon-like peptide-1 (GLP-1) receptor agonists and sodium-glucose-co-transporter-2 (SGLT-2) inhibitors) in the case of COVID-19 infection may be associated with a higher risk of complications, such as dehydration and ketoacidosis [30,31,32]. In the reports by Mota et al., it has been confirmed that in diabetic patients, using insulin therapy, a lower expression of ACE-2 was demonstrated [33]. Diabetic patients often require antihypertensive treatment because of concomitant hypertension, and angiotensin converting enzyme (ACE-I) inhibitors, angiotensin receptor blockers (ARBs) may cause overexpression of ACE-2 [34,35,36,37]. According to many researchers, the facilitated penetration of the virus into the cells of the human body is caused by increased expression of ACE-2 receptors in the cell membranes of the lung epithelium (type 2 alveolar cells) [38], intestines (enterocytes) [39], kidneys (proximal tubule cells) [34,35,40], as well as the heart muscle (myocardium cells) [36,37,41].

**Figure 3:**
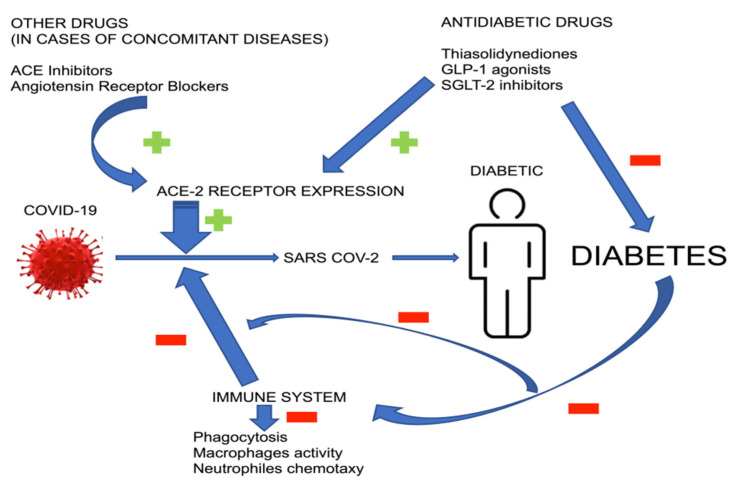
Patomechanisms inducing an increased possibility of COVID-19 infection in diabetic patients. Antidiabetic treatment, especially combined with other drugs, leads to overexpression of the ACE-2 receptor—a gateway for COVID-19. Deterioration of immune system function in diabetes facilitates the infection and increases its severity.

Diabetes predisposes patients to acquiring the infection due to an impaired immune system function. The following are responsible for this fact: the inhibition of phagocytosis, lower macrophage activity and decreased ability of neutrophil chemotaxis resulting from the reduced amount of interferon (IFN-γ) [42]. The role of glycaemic control in diabetic patients is emphasized. Although, as mentioned earlier [21], it does not seem to affect the efficacy of infection treatment, it reduces the likelihood of virus proliferation in cells [43,44]. Another possible cause of increased susceptibility to COVID-19 infection is an increase in the levels of furin, a type 1 protease bound to the cell membrane. It has been shown that higher levels of furin in diabetic patients facilitates the entry of SARS-CoV-2 into cells [33,45]. Furins mediate a viral entry into a human cell by cleaving and stimulating the SARS-CoV2 S1 and S2 proteins [3,46]. The scheme showing the pathophysiological mechanisms including an increased susceptibility of diabetic patients to COVID-19 is presented in Figure 3.

### 2.2. Immune System Disorders in Diabetic Patients

In type 1 and type 2 diabetes, there are changes in both the phagocytic activity of immune cells, which is reduced, as well as in the expression of cytokines. The level of pro-inflammatory mediators increases and the response based on the secretion of chemokines is suppressed [47]. Type 1 diabetes mellitus is characterised by a defect in the activation and function of regulatory T lymphocytes (Treg), leading to a sustained increase in the concentration of pro-inflammatory cytokines. In type 2 diabetes, an imbalance occurs in the activity of Th17 and Treg cells. Cytokines secreted by Th17 cells allow for the differentiation of obesity from obesity-related type 2 diabetes, while also stimulating the production of TNFα [48]. Thus, the increased secretion of proinflammatory cytokines in diabetics may change the nature of the immune response to SARS-CoV-2 infection into inflammation, increasing the probability of a severe course of COVID-19, leading to a cytokine storm, tissue and respiratory damage. Good glycaemic control may be important for maintaining the balance of the immune system, since lymphopaenia (30.5 vs. 49.6%) and neutrophilia (10.7 vs. 19.4%) compared to patients with high (>7.5 mmol/L) blood glucose levels [49].

### 2.3. Molecular Pathways Facilitating the Development of Hyperglycemia and Diabetes in COVID-19

#### The Influence of COVID-19 on Glucose Metabolism and the Pathomechanism of Diabetes

According to some researchers, more than half of COVID-19 patients experienced hyperglycaemia, and approximately 33% of patients developed diabetic ketoacidosis [50]. Marchand et al. were the first to observe an increased risk of developing type 1 diabetes in patients with COVID-19 [51]. The literature presents a case of a 29-year-old woman who was positive for glutamic acid decarboxylase-65 and negative for antibodies to tyrosine phosphatase IA2 as well as zinc transporter type 8 a month after infection with COVID-19. This preliminary report was later confirmed in a study on children with COVID-19 admitted to a hospital that had an increase in new cases of DM1 [52]. In recent studies, it has also been shown that COVID-19 infection can cause ketosis and ketoacidosis not only in diabetic patients [53]. Viral infections can induce the production of anti-pancreatic antibodies and, therefore, lead to the development of type 1 diabetes or other forms of insulin-dependent diabetes mellitus. The SARS coronavirus enters the cells of the pancreas via a functional ACE-2 receptor. After penetration, an autoimmune process is triggered, leading to the destruction of pancreatic islets and beta cells [54].

SARS-CoV-2 infection and the secondary reduction in insulin levels can contribute to both the induction of diabetes and the deterioration of its control in an existing disease [55]. According to another hypothesis, SARS-CoV-2 endocytosis leads to a decrease in ACE-2 activity, which causes an increase in angiotensin II (AngII) levels and vasoconstriction which, in turn, induces a significant reduction in the blood supply to the pancreas and β cells. This islet hypoperfusion leads to impaired insulin secretion [41]. Even in mild cases of COVID-19 infection, significant increases in cytokines, such as interleukin (IL) -6, IL-1β, and tumour necrosis factor-alpha (TNF-α) have been observed in severe comorbidities [56]. Infection with COVID-19 in diabetic patients also causes an increase in stress hormones, such as glucocorticoids, which can lead to hyperglycaemia [57]. Metabolic inflammation and excessive cytokine release in the course of SARS-CoV-2 infection among patients with previously-diagnosed insulin resistance may additionally disturb the carbohydrate metabolism, leading to the development of clinical diabetes [58]. Many authors emphasize the possibility of a two-way interaction between COVID-19 and diabetes [41]. Infection with SARS-CoV-2 can lead to diabetes mellitus and, conversely, diabetes increases the severity of COVID-19 infection [34]. In addition, prolonged hyperglycaemia can cause ACE2 pancreatic glycation and facilitate the viral entry into pancreatic B cells. This mechanism may worsen the course of COVID-19 [59]. It can be assumed that some of the symptoms of long-term COVID-19 infection, especially fatigue and general malaise, may be caused by deregulated metabolism and hyperglycaemia.

### 2.4. SARS-Cov-2 Related Complications

#### 2.4.1. Increased Risk of Cardiovascular Disease

In the course of diabetes mellitus, micro- and macrovascular complications occur, which include cardiovascular diseases. It has been found that diabetic patients with COVID-19 are at increased risk of their development. The Wuhan reports allow us to directly confirm the above conclusion [60]. According to one of them, as many as 32% of hospitalised patients had chronic diseases, the most common of which was diabetes [60]. Viral pneumonia was the most common manifestation of COVID-19, but cardiovascular symptoms predominated in some cases [61]. The most frequent of these are myocardial damage, arrhythmias, acute coronary syndrome and thromboembolism [62]. Cardiovascular complications usually occur sometime after the acute phase of the disease [63]. 

In a recent study by Zou et al., it was shown that ACE-2 receptors are present in over 7.5% of myocardial cells, the presence of which may facilitate the entry of the virus into cardiomyocytes and, consequently, lead to cardiotoxicity [64]. Also, in the analyses based on the reverse transcription polymerase chain reaction (RT-PCR), SARS-CoV-2 molecules in the myocardium were detected [65]. Certain pro-inflammatory cytokines, such as TNF-α, IL-1, and IL-6, may also be responsible for inducing a negative inotropic effect on the heart [66].

#### 2.4.2. Oxidative Stress

Diabetes mellitus is a systemic disorder characterised by chronic hyperglycaemia, directly resulting from inflammation and oxidative stress [67]. Oxidative stress is a state of disturbed balance between the by-products of metabolic changes, i.e., reactive oxygen species, and the ability of tissues to remove them from the body, i.e., detoxification. This imbalance may play a significant role in the pathogenesis of SARS-CoV-2 infection [67]. Reactive oxygen species (ROS) and reactive nitrogen species (RNS) are products of various cellular metabolic processes. NADPH oxidase is a complex of five proteins encoded by the CYBA, CYBB, NCF1, NCF2 and NCF4 genes. The enzyme is located in the cell membrane of phagocytes, where it takes part in an oxygen explosion, during which a superoxide radical with bactericidal properties is produced. Mutations of any of the NADPH oxidase genes cause chronic granulomatous disease, characterised by recurrent bacterial infections as well as an increased predisposition to cancer. NADPH oxidase is also found in other cell types, including B-lymphocytes, where reactive oxygen species act as signalling molecules involved in cellular processes such as differentiation and proliferation [68]. The binding of ACE-2 to the viral S protein leads to ACE-2 dysfunction and an increase in the concentration of angiotensin II, which binds the type 1 angiotensin receptor (AT1R) and stimulates NADPH activity. This further leads to the increased production of ROS (peroxides) and the consumption of NADPH. It can be concluded that NADPH oxidase activity may be increased in the case of SARS-CoV-2 infection and results in increased cellular oxidative stress. Also, lower glutathione levels increase oxidative stress and lead to immune dysfunction as well as greater susceptibility to SARS-CoV-2 infection [69].

#### 2.4.3. Coagulation Disorders

One of the most common complications of COVID-19 infection is a disorder in blood clotting. Thrombotic events were diagnosed in 16–49% of patients admitted to intensive care units [70]. Diabetes mellitus is associated with decreased fibrinolytic activity which causes an increase in the activity of the coagulation system. Fibrinolysis is the final stage of haemostasis during which the thrombus is broken down. Similarly to blood clotting, it is a cascade process. The key enzyme for fibrinolysis is plasmin which is formed from plasminogen. Weakened intensity of fibrinolysis in diabetes mellitus is associated with an increase in the concentration of many antifibrinolytic proteins and some changes in the fibrin network [71]. As in other severe infections, in the case of COVID-19, factors responsible for the increase in blood clotting are taken into account: stasis due to immobilisation, excess inflammatory cytokines and endothelial dysfunction. Most disseminated intravascular coagulopathies are associated with an increase in the activity of coagulation factors and an increased concentration of D-dimers in the blood serum. Interestingly, in the case of COVID-19, the concentration of D-dimers increases, but the activity of clotting factors remains within the laboratory range, which may indicate that platelet activation and dysfunctional fibrinolysis are responsible for COVID-19-related coagulopathy [72]. Increased D-dimer levels in patients with COVID-19 may be due to inflammatory responses to viral infections and the dysfunction of endothelial cells that increases thrombin production. In these patients, the existence of a concomitant disease such as diabetes, cancer, stroke, and a physiological condition such as pregnancy can also lead to higher levels of D-dimer [73].

It should be emphasized that diabetes is also a state of “atherothrombosis” associated with platelet activation, endothelial dysfunction and plaque formation [74]. The processes described above are responsible for an increased risk of cardiovascular events in people with diabetes.

#### 2.4.4. Hypoxia

Infection with SARS-CoV-2 leads to the development of both pulmonary and extrapulmonary complications. Initially, SARS-CoV-2 enters the body through the respiratory tract and binds to the ACE-2 receptor on AT2 cells in the alveolar epithelium, and then enters them. This causes the development of respiratory symptoms. When attacked by the virus, AT2 cells produce inflammatory mediators and stimulate the production of pro-inflammatory chemokines as well as cytokines. This process leads to endothelial dysfunction, causing blood vessels to dilate and induce inflammation within their walls, resulting in diffuse interstitial exudate. The next stage in this cascade of events is pulmonary oedema leading to alveolar gas dysfunction, which is known as acute respiratory distress syndrome (ARDS) [75]. Chronic hyperglycaemia can lead to interstitial fibrosis and alveolar capillary microangiopathy which can cause both restrictive and obstructive pulmonary dysfunction. There is a reduction in forced expiratory volume during the 1st second (FEV1), forced vital capacity (FVC), diffusion lung capacity for carbon monoxide (DL) and elasticity lung tissue [76]. Lung function impairment in diabetes mellitus may be associated with an increased risk of ARDS in the presence of SARS CoV-2 infection. Approximately 30% of patients with diabetes and COVID-19 develop lung dysfunctions, as evidenced by a decline in the FEV1/FVC ratio. Significantly impaired lung function leads to chronic hypoxia of the heart and brain [77]. Observations have shown that cardiovascular complications in COVID-19 occur in 1/3 of cases and include myocardial damage, arrhythmias, sudden cardiac arrest, heart failure and coagulation disorders [77].

#### 2.4.5. Cytokine Storm

“Systemic hyper-inflammation”, known as a cytokine storm, is often responsible for the severe course of disease and the rapid deterioration of the clinical condition. It is a state of uncontrolled systemic inflammation caused by excess cytokines and leads to multiple organ failure and sometimes even death. Various cytokines are involved in its development, including the IL-1, IL-6, IL-8, IL-10, TNF-α and IFN-γ families [78]. McGonagle et al. concluded that the cytokine storm in COVID-19 is the result of the inability of the immune system to clear the virus. Researchers divided its course into two stages. The first is similar to haemophagocytic lymphohistiocytosis and is associated with immunodeficiency. It is characterised by a delayed secretion of interferons I and III. This leads to an excessive delayed immune response and severe COVID-19 as manifested, inter alia, in generalised pneumonia, followed by the development of ARDS. The second is an overactive immune state that aims to compensate for the primary failure to clear the virus. It is based on the excessive secretion of pro-inflammatory cytokines from mononuclear macrophages. This sequence of events results in the clinical manifestation of a cytokine storm [79]. Why some people develop a cytokine storm in COVID-19 and others do not remains an issue requiring further research. A potential cause may be genetic predisposition to its occurrence. This hypothesis may be supported by the phenomenon of a genetic predisposition to cytokine storms in primary haemophagocytic syndrome (HLH), which results from genetic defects in the granzyme and perforin-dependent pathway of cytotoxic T lymphocytes and NK cells [80]. In diabetic patients, an increase in the number of activated neutrophils and the formation of neuroendocrine tumours (NETs) may be associated with the phenomenon of cytokine storms and severe COVID-19 infection [81]. One recently discovered early prognostic factor for severe COVID-19 and a cytokine storm is increased neutrophil to lymphocyte ratio [82].

#### 2.4.6. COVID-19 and Pancreatitis

The post-mortem examination of COVID-19 patients showed a higher proliferation of SARS-CoV-2 in the pancreatic tissue. The pancreas has a large number of ACE-2 receptors mainly located in β cells and the exocrine ducts [83]. The binding of SARS-CoV-2 to pancreatic ACE-2 receptors stimulates metalloprotease-17 (ADAM-17) and disintegrin, sequentially activating ACE-2 receptors and increasing TNF-alpha production [84]. Taneera et al. demonstrated that SARS-CoV-2 influences the activity of pancreatic lipase and affects peripheral adipose tissue, causing pancreatic damage and induction of a cytokine storm [85]. Thus, damage to the pancreas in COVID-19 can occur in two ways, either by direct invasion of SARCoV-2 or indirectly by inducing a cytokine storm [86].

#### 2.4.7. Central Nervous System Complications

Neurological manifestations of COVID-19 are not only associated with a direct influence of the SARS-CoV-2 on the nervous system but also with some immune-mediated diseases during infection or even after recovery, and with some neurological complications of the systemic effects of COVID-19. 

The Chinese researchers Mao et al., observed CNS symptoms in 25% of hospitalized patients due to COVID-19. Among these symptoms, the most common were dizziness (17% patients), headache (13%), and impaired consciousness (7%) [87]. Guillain–Barre Syndrome (GBS) associated with SARS-CoV-2 infection has also been described [88]. Cases of possible COVID-19-related encephalitis have also been reported [89,90,91]. Another neurological manifestation of a SARS-CoV-2 infection may be acute disseminated encephalomyelitis, which is a syndrome of multifocal demyelination and usually occurs weeks after infection [92].

In one national registry out of 125 patients with COVID-19 and neurological diseases, 77 (62%) patients had a cerebrovascular event: 57 had (46%) ischaemic strokes, nine (7%) had an intracerebral haemorrhage and one had central nervous system vasculitis [93].

Neurological complications, although uncommon compared to other complications such as those of the respiratory system, especially encephalitis and stroke, can cause lifelong disability with associated long-term care needs and potentially high health and economic costs.

Further carefully designed clinical and epidemiological studies are needed to help identify symptoms and understand the pathomechanism of SARS-CoV-2-induced neurological diseases.

### 2.5. Pharmacotherapy in COVID-19

The currently applied course of pharmacotherapy in patients with COVID-19 may have a significant impact on glycaemia. Corticosteroids which, on the one hand, reduce the excessive immune response and, on the other, increase blood glucose levels can serve as an example. Particularly effective are high doses of methylprednisolone (80–160 mg/day for seven days) used in the treatment of COVID-19, which may cause serious clinical hyperglycaemia. For dexamethasone, a reduction in mortality over 28 days of treatment was shown in the group of patients receiving oxygen or invasive mechanical intervention. Therefore, it is currently recommended for the treatment of the most severe form of COVID-19. From a clinical point of view, it is worth monitoring glycaemia closely in patients with COVID-19 treated with steroids to prevent complications caused by hyperglycaemia [94]. Chloroquine and hydroxychloroquine have been shown to be effective in inhibiting SARS-CoV-2 replication, but may also trigger a cytokine storm. Hydroxychloroquine stimulates the proper activity of beta cells, insulin secretion, and improves peripheral glucose metabolism. Thus, the treatment with hydroxychloroquine in COVID-19 may help to improve diabetes control. However, it can often lead to the occurrence of serious hypoglycaemic episodes. During its use, special care should be taken and glycaemia monitored [95].

#### 2.5.1. Hypoglycaemic Therapies in Patients with Diabetes and COVID-19

Most authors believe that insulin should be the drug of choice in hospitalised COVID-19 patients with co-existing diabetes, because the use of insulin therapy in these patients satisfactorily lowers blood glucose levels. The American Diabetes Association recommends a continuous intravenous infusion of insulin in critically ill patients as well as the implementation of subcutaneous insulin or correction of basal rate (possibly, as a bolus) for hospitalised patients in stable condition. In both groups of patients, it is recommended to aim at glycaemia within the range of 7.8–10.0 mmol/L. It has been suggested that continuous insulin infusion may not only be an effective method of achieving glycaemic control, but it can also improve the treatment outcomes in patients with hyperglycaemia and COVID-19 [96]. Interestingly, Chen et al. reported an over three-fold increase in the risk of death and severe prognosis among hospitalised diabetic patients with COVID-19 using insulin therapy. As such, it remains unclear whether insulin therapy improves or worsens COVID-19 outcomes. The analysis of these studies should also take into account bias in the selection of patients, i.e., those with diabetes treated using insulin are usually characterised by a longer duration of the disease and a higher percentage of comorbidities [97].

#### 2.5.2. Glucagon-Like Peptide-1 Receptor (GLP-1R) Agonists

Glucagon-like peptide-1 (GLP-1) is an incretin hormone produced by the endocrine cells of the intestine. Pharmaceuticals based on GLP-1 are used to treat diabetes and act through the GLP-1 receptor (GLP-1R), which is present in the lung epithelium, as well as in some immune cells. GLP-1-based drugs have beneficial effects on inflammation-induced acute lung problems. They stimulate the secretion of the atrial natriuretic peptide (ANP), a peptide natriuretic hormone known as a potent pulmonary vasodilator. They inhibit the activity of many cytokines and chemokines in the lungs and act anti-inflammatorily by reducing the concentration of the thioredoxin interacting protein (TxNIP) while increasing the activity of nitric oxide synthase (eNOS). Considering the mechanism of the action with regard to GLP-1R agonists, it should be hypothesised that COVID-19 patients could benefit from this type of therapy, but this requires clinical trials [98].

#### 2.5.3. SGLT-2 Inhibitors

In the course of COVID-19, an increased activity of lactate dehydrogenase (LDH) was found, and thus, an increased risk of lactic acidosis. Dapagliflozin, an inhibitor of the sodium-glucose transporter-2 (SGLT-2), may be beneficial because it reduces glycaemia through various mechanisms. SGLT-2 inhibitors impede the Na+/H+ (NHE) exchanger, where they then protect against swelling and cell disintegration. Moreover, SGLT-2 inhibitors lead to a reduction in the concentration of pro-inflammatory cytokines, including those directly related to the “cytokine storm”. They also increase the expression of ACE-2, leading to the activation of the alternative renin-angiotensin-aldosterone pathway, which is disturbed in SARS-CoV2 infection [99]. In light of the cited reports, it was decided to conduct an international, double-blind, randomised, phase III, placebo-controlled trial—DARE-19 (Dapagliflozin in respiratory failure in patients with COVID-19—clinical trial number NCT04350593). The study included hospitalised patients with COVID-19 with at least one cardiometabolic risk factor (hypertension, type 2 diabetes, atherosclerosis, heart failure or chronic kidney disease). In the study, it was concluded that in patients with cardiometabolic risk factors who were hospitalised for COVID-19, treatment with dapagliflozin did not result in a statistically significant reduction in the risk of organ dysfunction or death, or in an improvement in clinical health, but it was well-tolerated. The panel of experts advises against the use of SGLT-2 inhibitors among COVID-19 patients due to the risk of dehydration and euglycaemic ketoacidosis [100].

Table 2 shows the pleiotropic effect of hypoglycemic drugs on the cardiovascular and coagulation systems. This influence can lead to favorable or unfavorable changes that have a significant impact on the course of SARS-CoV-2 infection in diabetics. In most cases, these drugs reduce the prothrombotic risk characteristic of the course of SARS-Cov2 infection [101].

The benefits, risks and recommendations for anti-diabetic treatment are presented in Table 3 [102].

#### 2.5.4. Vaccination for COVID-19 in Patients with Diabetes Mellitus

Due to the increased risk of a severe course of COVID-19 in the group of diabetics, primary prophylaxis in the form of vaccinations is essential. The humoral immune response against SARS-CoV-2 in patients with DM was comparable to that in patients without diabetes. Importantly, glycaemia does not affect this. The presence of IgG antibodies against the viral receptor binding domain (RBD) was a prognostic factor in the survival rate among both diabetic and non-diabetic patients [103]. Timely vaccination plays an extremely important role in the primary prevention of COVID-19 infection.

## 3. Conclusions

SARS-Cov-2 infection impairs glycaemic control in diabetes by increasing inflammation and altering the nature of the immune system’s response. Coronavirus infection increases the risk of complications in diabetic patients, leading to the development of thromboembolism or cardiovascular and respiratory failure. More research is needed to determine the optimal management of patients with diabetes and COVID-19. It seems particularly important to promote vaccination against COVID-19 in this group of patients.

## Figures and Tables

**Table 1:** Overview of risk regarding adverse COVID-19-related outcomes according to glycaemic control.

Study	Publication Country and Year	Type of Study	Overall Population	DM Type 1 Population	DM Type 2 Population	Glycaemic Control	Mortality
Barron et al. [23]	United Kingdom, 1 March–11 May 2020	nationwide population-based cohort	3,128,500	263,830	2,864,670	-	T1D 3.51 (3.16–3.90) T2D 2.03 (1.97–2.09)
Holman et al. [24]	United Kingdom, 1 March–11 May 2020	nationwide population-based cohort	3,154,300	265,090	2,889,210	HbA1c 59–74 mmol/mol	T1D 1.16 (0.81–1.67) T2D 1.22 (1.15–1.3)
						HbA1c 75–85 mmol/mol	T1D 1.37 (0.9–2.07) T2D 1.36 (1.24–1.5)
						HbA1c ≥ 86 mmol/mol	T1D 2.23 (1.5–3.3) T2D 1.61 (1.47–1.77)
Wang et al. [25]	China, 24 January–10 February 2020	multi-centre retrospective cohort	605	-	-	Fasting blood glucose level ≥ 7 mmol/L (126 mg/dL)	HR 2.30 OR 3.99
Wu et al. [26]	China, 26 December 2019–15 March 2020	multi-centre retrospective cohort,	2041	-	-	Hyperglycaemia ≥ 6.1 mmol/L (110 mg/dL)	HR 1.30
Copelli et al. [27]	Italy, 20 March–6 April 2020	single-centre retrospective cohort	271	-	-	Hyperglycemia ≥ 7.78 mmol/L (140 mg/dL)	HR 1.80
Bode et al. [28]	USA, 1 March–6 April 2020	multi-centre retrospective cohort	1122	-	-		OR 6.12
Zhu et al. [29]	China, 30 December 2019–20 March 2020	multi-centre retrospective cohort	7337	-	952		HR 0.14
Williamson et al. [14]	United Kingdom, 1 January– 6 May 2020	nationwide population-based cohort	17,278,392	-	-		OR 2.61 HR 1.95
Cariou et al. [21]	France, 10 March–31 March 2020	multi-centre cohort	846	-	-	HbA1c 53–63 mmol/mol	OR 1.55
						HbA1c 64–74 mmol/mol	OR 1.09
						HbA1c ≥ 75 mmol/mol	OR 0.84

COVID-19—coronavirus disease 2019, T1D—type 1 diabetes mellitus, HR—hazard ratio, OR—odds ratio.

**Table 2:** Pleiotrophic effect of hypogycemic drugs on the cardiovascular and coagulation system.

Drug	Cardiovascular	Effect		Coagulation	Effect	
		↑	↓		↑	↓
Acarbose	Risk of cardiovascular events		x	PLT—bound fibrinogen		x
	Progression of carotid intima–media thickness		x	P—selectin platelet exposure		x
				Platelet—monocytes aggregates formation		x
Dipeptyl peptidase-4 inhibitors	Benefit on MACE	- -		PLT activation and oxidative stress markers		x
				cAMP formation and PKA activation	x	
				Plasma fibrinogen and PAI-1		x
				Soluble levels of CD40		x
				Inflammatory and thrombogenic gene expression		x
				Platelet mitochondrial respiration and aggregaton	x	
				Intracellular free calcium and tyrosine phosphorylation leading to PLT aggregation		x
GLP-1 receptor agonists	MACE and fatal or non-fatal MI		x	Thrombin, ADP, and collagen—induced PLT aggregation mediated by cAMP—induced PKA activation and increased eNOS enzymatic activity		x
				ROS production		x
				cGMP production	x	
				VASP-ser239 phosphorylation	x	
				PI3-K/Akt and MAPK/erk-2 pathways		x
				NO bioavaliability	x	
				ROS production		x
				Platelet P-selectin expression		x
Metformin	MI, stroke and all-cause mortality		x	ADP, collagen and arachidonic acid induced platelet aggregation		x
	Macrovascular complicatrions (MI, stroke, peripheral vascular disease)		x	Production od superoxide ion (O2-)		x
				PLT activation and axtracellular mitochondrial DNA release		x
				11-dhTXB2 urinary excretion		x
				8-iso-pg F2 α excretion		x
				Mean PLT volume		x
Sodium-glucose cotransporter 2 inhibitors	Incidence of MACE, cardiovascular death and hospitalization for HF		x	ADP—induced PLT activation		x
				P selectin mRNA expression		x
				ROS bioavaliability		x
				NO bioavaliability	x	
				Advanced glycation end products		x
				e NOS activation	x	
				Interstitial and periarterial NO stress		x
Sulphonyloureas	Cardiovascular benefit vs. metformin alone		x	ADP-induced PLT activation		x
	Risk of hospitalization/mortality	x		PLT adhesiveness		x
	Risk of stroke and overall mortality	x		Oxidative stress		x
	MACE	- -	- -	Cyclooxygenase and lipoxygenase pathways		x
	All—cause mortality, cardiovascular mortality, MI or stroke with 2nd or 3rd generation drugs					
Thiasolidynediones	Pioglitasone—MACE	-	-	ADP-induced PLT aggregation		x
	Pioglitasone—MI/stroke		x	P selectin levels		x
	Rosiglitasone-Risk of cardiovascular events	x		Inflammation and macrophage recruitment		x
	Rosiglitasone-MI/cardiovascular death	-	-	E-selectin		x
	Rosiglitasone-HF hospitalisations	x		vWillebrand, SCD40L, PAI-1, 11-dhTXB2		x

ADP—adenosine diphosphate, ERK—extracellular signal-regulated kinases, eNOS—endothelial nitric oxide synthase, GLP-1 glucagon like peptide-1, HF—heart failure, MAcEs-major cardiac adverse events, MAPK—mitogen- activated protein kinases, MI—myocardial infarction, NO—nitric oxide, PAI-1—plasminogen activator inhibitor-1, PI3K—phosphatidyl inositol-3 kinase, PKA—protein kinase A, PLT—platelet, ROS—reactive oxygen species, TXB—thromboxane, VASP—vasodilator-stimulatedphosphoprotein, ↑—increase, ↓—decrease.

**Table 3:** Benefits, risks and recommendations for anti-diabetic treatment with COVID-19 and diabetes mellitus.

Pharmacotherapy	Benefits	Risks	Recommendations
Insulin	Precise dosing possible for better glucose control. Better COVID-19 outcomes.	Increased risk of hypoglycemia (special care should be taken in combination with chloroquine therapy). High insulin requirements in critically ill patients.	Indicated in severe COVID-19.
Metformin	Anti-inflammatory activity.	Increased risk of lactic acidosis especially in critically ill patients.	Contraindicated in severe COVID-19.
SGLT-2 inhibitors (Sodium-glucose-co-transporter-2)	No hypoglycemic effect.	Increased risk of dehydratation and ketoacidiosis	Contraindicated in severe COVID-19.
Sulfonylureas	Not applicable	Increased risk of hypoglycemia (special care should be taken in combination with chloroquine therapy).	Not recommended in severe COVID-19 and increased risk of hypoglycemia.
GLP-1 receptor agonists (Glucagon-like peptide 1)	No hypoglycemic effect, anti-inflammatory activity	Increased risk of dehydratation	It is possible to continue the therapy provided an adequate fluid intake and regular meals are consumed
Thiazolidinediones	Insulin resistance reduction, anti-inflammatory activity	Increased risk of fluid retention.	Not recommended in severe COVID-19 and heart failure.
DPP-4 inhibitors (Dipeptidyl peptidase 4)	No hypoglycemic effect, good treatment tolerance	Not applicable	Continuation of treatment indicated

## Data Availability

Not applicable.
